# Prevalence and associated factors with mental health symptoms among semi-professional cricket players after the resumption of sporting activities following an extensive lockdown

**DOI:** 10.17159/2078-516X/2023/v35i1a15058

**Published:** 2023-06-05

**Authors:** L Malele, H Noorbhai

**Affiliations:** Department of Sport and Movement Studies, Faculty of Health Sciences, University of Johannesburg, South Africa

**Keywords:** depression, anxiety, psychology, cricketers, COVID-19

## Abstract

**Background:**

Mental health of cricket players has been a topic of debate for a considerable time across the globe.

**Objectives:**

The purpose of this study was to investigate the prevalence of mental health symptoms among semi-professional male cricket players experienced during COVID-19, as well as the relationship between age and the Depression, Anxiety, Stress Scale (DASS-21) sub-scale.

**Methods:**

Mental health symptoms were assessed among cricket players (n = 90) using the following instruments, respectively: Depression, Anxiety, Stress Scale – 21 (DASS – 21); Athlete Burnout Questionnaire (ABQ) and Satisfaction with Life Scale (SWLS). Descriptive (means ± standard deviations) and inferential (Spearman’s correlations) statistics were calculated using SPSS (IBM Version 27.0) at a significance level of p < 0.05.

**Results:**

The study reported that 5.6% (n = 5) of cricketers believed life was futile, and 10% (n = 9) thought they were useless most of the time. However, on the anxiety sub-scale, 27% (n = 24) of cricketers indicated low confidence. In addition, 23% (n = 21) of cricketers reported being stressed. Spearman's correlations revealed a positive and significant association between the DASS-21 sub-scales and that cricketers' DASS-21 sub-scale symptoms are unrelated to age.

**Conclusion:**

The study found that there were moderate levels of anxiety, a reduced sense of achievement and a neutral feeling towards satisfaction with life. Reducing mental health symptoms would extend the playing careers of cricket players. De-stigmatising mental health may result in more robust and accurate self-reports of mental health illness among elite athletes, which can enable sustainable change.

Cricket is considered one of the most demanding team sports in terms of athleticism and skill. ^[1]^ In addition to the physical load, it also carries high psychosocial stress that affects the player both during and after the game. ^[1, 2]^ Even though more cricket players are revealing what they experience, mental health illness is still stigmatised. ^[2, 3]^ The mental health of cricket players across the globe has been a topic of debate for a considerable time. ^[1, 2, 3]^ The athlete's mental status, similar to their physical status, can be viewed as a resource that allows players to cope with the pressures of the game ^[4]^ The reluctance of cricket players to speak freely about their mental health demonstrates inadequate access to mental health care in industrialised and especially, underdeveloped nations. ^[1]^ Several studies have been published so far on the topic of cricket and mental health. ^[2,4,5]^

Ogden et al. conducted a qualitative investigation of mental health among professional cricket players in the United Kingdom. They documented that perceived mental health stigma can be reduced by providing stability with player contracts, helping players prepare for transitions, encouraging healthy habit development, as well as optimal coach relationships and communication. ^[4]^ Similar to the present study, Hendricks et al. conducted a cross-sectional survey design to determine the prevalence of anxiety and depression symptoms in professional cricket players, as well as to identify factors associated with symptoms of anxiety and depression. ^[5]^ Their study showed that the prevalence of symptoms of anxiety and depression was 59% and that both the contributing and protective factors were career-related, which can be modifiable. ^[5]^ Both studies have suggested that further research is required to help build a greater base of understanding of mental health in cricket in order to assist with the development of interventions, maximise the impact of psychological practice within cricket, as well as the inclusion of associated factors into mental health literacy programmes. ^[4,5]^

During the pandemic, athletes’ lifestyles were disrupted, which led to additional mental health stressors. ^[3]^ Changes in the way we have adjusted to the new normal (digital technologies, lessons from lockdown, etc.) and the inability to train normally are just some of the challenges athletes have had to endure due to the COVID-19 pandemic. ^[6]^ According to DASS–21, an increase in certain emotions, such as anxiety, can result in improved performance. ^[8]^ In addition, the importance of self-awareness and continuous mental health promotion among elite athletes is undervalued. ^[9]^ Sambo athletes benefit from physical activity by feeling better and enhancing their mental health. ^[10]^ The sambo athlete’s life satisfaction (SWLS) increases as the athlete engages in physical activity.

Since cricketers' mental health has received little attention, this is a gap that needs to be explored further. Internal structures of sports organisations may emphasise mental toughness as critical for athletes, but they may fail to establish spaces where elite athletes can talk about their personal mental health difficulties to foster an open dialogue about the focal issue. ^[2]^ Studies on athletes' mental health (and their symptoms) could lead to new ways to assist athletes who have been suffering with mental health challenges.

As a result of the limited studies conducted on mental health in cricket (which has been further highlighted by the COVID-19 pandemic), as well as the numerous accurate tools that are available for an athlete’s mental health, the objectives of this study are to: (i) investigate the prevalence of mental health symptoms (anxiety, depression, stress, physical and emotional exhaustion (PEE), devaluation of sports practice (DSP), reduced sense of accomplishment (RSA) and satisfaction with life (SWLS)) among semi-professional male cricket players experienced during COVID-19; and (ii) investigate the relationship between age and depression, anxiety and stress, as well as how they affect one another.

## Methods

### Study design

A cross-sectional survey design was employed in this research study. The study obtained ethical clearance from the Faculty of Health Sciences Research Ethics Committee (REC-1130-2021) at the University of Johannesburg.

### Setting

The research was conducted across different leagues in the Western Cape of South Africa, namely: Western Province (WP) Premier League (AMA 20), WP First Division A (AMA 20), WP First Division C (AMA 20) and WP Second Division. Both the Varsity College cricket club and the Western Province cricket club were assessed on the same cricket ground, as they share the same venue. Wynberg, Green Point and Milnerton cricket clubs were all tested at their respective grounds. The survey design was utilised to identify the prevalence of mental health symptoms among cricket players after the resumption of sporting events following an extensive lockdown (between September 2021 and May 2022) as a result of COVID-19.

### Participants

The inclusion criteria were male semi-professional Western Cape cricket players (provincial B and university squads), with an age range from 18 to 35 years, who had no chronic injuries or mental health illnesses. The initial sample size that was required was 150 (confidence level of 95%, within ± 5% of the measured value). The final sample size obtained for the study was 90 cricket players. For definition purposes, a semi-professional cricket player refers to a cricket player who plays for a first-class team but not for a professional county/state/franchise team. ^[11]^

### Outcome measures

#### Depression, Anxiety, Stress Scale – 21 (DASS–21)

A total of seven items were included in each of the three DASS-21 scales. These sub-scales were depression, anxiety and stress. The depression scale evaluates symptoms such as dysphoria, hopelessness, a low opinion of oneself, a lack of enthusiasm or participation, anhedonia and laziness. The anxiety scales include measures of autonomic arousal, skeletal muscle effects, situational anxiety and subjective feelings of anxiousness. The stress scale has an effect on the level of persistent non-specific arousal. The following are the recommended cut-off scores for traditional severity labels: normal, moderate and severe. ^[12]^ The recommended cut-off scores for conventional severity labels (normal, moderate, severe) and scores on the DASS-21 sub-scales will need to be multiplied by 2 to calculate the final score.

#### Athlete Burnout Questionnaire (ABQ)

The ABQ is a 15-item scale that measures the level of athlete exhaustion. In the questionnaire for athletes with burnout ^[13]^, the condition was characterised by a combination of physical and emotional exhaustion (PEE), devaluation of sports practice (DSP) and reduced sense of accomplishment (RSA). Cricket players ranked the frequency of their experience on a five-point Likert scale with 1 = almost never, 2 = seldom, 3 = occasionally, 4 = frequently and 5 = very constantly. Raedeke and Smith reported internal consistency estimates of 0.91 for emotional/physical exhaustion, 0.85 for impaired sense of accomplishment, and 0.90 for devaluation. ^[13]^ The grading method is based on the participant's average number of replies and ranges from 1 to 5.

#### Satisfaction with Life Scale (SWLS)

The SWLS holistically assesses the athlete’s cognitive judgement of life. ^[14]^ The test comprises of five questions, which are answered on a scale from one to seven. The SWLS is an interval scale. Scores from 1 to 1.86 indicate a considerable disagreement. Categorisation of the cricket players’ responses followed the Pimentel study ^[15]^ and were namely, strongly disagree (1.00 to 1.86), slightly disagree (1.86 to 2.71), somewhat disagree (2.71 to 3.57), neither agree nor disagree (3.57 to 4.43), somewhat agree (4.43 to 5.29), slightly agree (5.29 to 6.14) and strongly agree (6.14 to 7.00).

These three questionnaires were used in similar studies: DASS-21 ^[8]^, ABQ ^[16]^ and SWLS. ^[10]^

### Study procedure

The recruitment process included sending coaches and club managers study information via digital and telephonic communication. The coaches and managers were approached first, as it was required to obtain permission to invite players if they would like to participate in the study. The players were subsequently invited and also informed about the research, as well as what was expected of them. Informed consent was signed by the cricket players before completing the questionnaires. The study goals and any potential undesirable effects were described in detail. Three mental health surveys were completed (between September 2021 and May 2022) by cricket players via an electronic link. The survey was designed in a way so that in order to proceed to the next question or section, the cricket player needed to complete the current question or section. In so doing, it eliminated any blank spaces or risk for non-responsiveness. Participants were able to go back and change answers if needed. The player would be referred to a psychologist if they reported a severe rating according to the cut-off scores of the questionnaires.

### Statistical analysis

All of the survey questions were fixed responses, quantitative in nature. All results were gathered in real-time using an online survey platform (Google Forms), and exported to Microsoft Excel (2021) for analysis. For the first objective, descriptive statistics were employed to determine the prevalence of mental health symptoms. For the second objective, Spearman's correlations were employed to determine the relationship between DASS-21 and age. The Statistical Package for the Social Sciences (SPSS, IBM Version 27.0) for Windows was used to perform the statistical analysis on the data at a significance level of p < 0.05.

## Results

### Participants

Due to COVID-19, the sample composition had to be altered based on the availability of cricketers and physical distancing. The average age of all participants was 24 ± 5 years. The selected sample consisted of five different teams: Western Province Cricket Club (18 %), Green Point Cricket Club (27%), Milnerton Cricket Club (27%), Wynberg Cricket Club (21%) and Varsity College Cricket Club (8 %). Most respondents were all-rounders (47 %), followed by bowlers (27 %), batters (18 %) and wicket-keepers (9 %).

### Depression, anxiety, and stress indicators

**Fig. 1:**
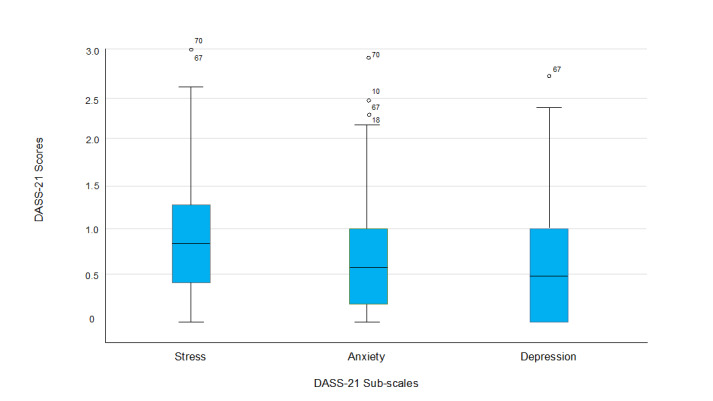
DASS-21 based on sub-scales (depression, anxiety and stress) and how cricket players responded (n=90). Box indicates the median and interquartile range; whiskers indicates the range. The circles and numbers on the figure represent the outliers (e.g. player 70).

A non-parametric Spearman’s correlation indicated that there was a strong, positive and significant correlation between stress and anxiety (r = 0.79; p = 0.000); stress and depression (r = 0.77; p = 0.000); and anxiety and depression (r = 0.75; p = 0.000). The survey revealed a trend that when one variable increases, so does the other. There were outliers reporting that some cricket players had elevated symptoms of either depression, anxiety or stress (Figure 1). The numbers on the figure represent the players (e.g. 70).

Table 1 shows that symptoms were evident in each of the three DASS-21 sub-scales: Depression (4.37 ± 5.97) at a high normal level; Anxiety (5.03 ± 6.37) at a moderate level; Stress (6.82 ± 6.48) at a moderate level. There was no statistically significant difference between the positions of cricket players. According to Spearman’s correlation, the relationship between DASS-21 and age was weak, negative, and not significant as is evident in Table 1. The relationship between age and anxiety (r = − 0.168; p = 0.114); and age and depression (r = − 0.052; p = 0.625) was not significant. A slight variation was found in the relationship between stress and age (r = 0.058; p = 0.585) which was weak, positive and not significant.

### Physical and emotional exhaustion (PEE), devaluation of sports practice (DSP), and reduced sense of accomplishment (RSA)

Table 1 shows the ABQ variables that were measured: PEE (2.17 ± 1.03) with 10% of the participants feeling physically worn out by the sport; and 20% exhausted by the mental and physical demands of cricket during the season; DSP (1.89 ± 1.06) was a rare occurrence; and participants sometimes experiencing a reduced sense of achievement (RSA; 2.99 ± 0.97).

### Life satisfaction

According to the study, 14% of participants were dissatisfied with their current life circumstances. Overall, they neither agreed nor disagreed (4.46 ± 1.61) about being satisfied with life (Table 1).

## Discussion

The objective of this study was to investigate the prevalence of mental health symptoms (anxiety, depression, stress, PEE, DSP, RSA and SWLS) experienced among semi-professional male cricket players during COVID-19. A secondary objective of this study was to investigate the relationship between age and the DASS-21 sub-scale, as well as how they affect one another. The study found that mental health problems were less common among cricket players in lower divisions and that they were less likely to be prone to anxiety or depression. It has been found that 23% of cricketers had a difficult time winding down and that a variety of mental health difficulties, including mood disorders, suicide, drug and alcohol abuse and depression have been associated with cricket-related challenges. ^[1, 4]^

### Mental health research among athletes

In South Africa, only 5% of the national budget is allocated to mental health and only 50% of hospitals have the capability to deal with mental health conditions. ^[7]^ There is limited data on mental health illness among semi-professional athletes ^[10]^, as more studies relate to elite or professional athletes. Studies have shown that male athletes tend to have low response rates when it comes to speaking out or filling out questionnaires. ^[4]^ The analysis from this study reported that all the DASS-21 sub-scales were interrelated, and this is in line with Ali and Green. ^[17]^ Results in this study support the use of DASS-21 in a sports context, thereby, providing researchers with a reliable and valid mental health testing tool.

### Mental health research among semi-professional cricket players

Limited research exists on the mental health profiles of cricket players and athletes as a whole. ^[1, 4]^ In the study, only 3.3% were unaware of changes in somatic anxiety. Levels of anxiety were expected to be high due to the pandemic and uncertainty of what will happen next. ^[3]^ A study by Tubic et al. ^[10]^ reported that semi-professional athletes were not equipped to handle high arousals of symptoms of anxiety. In contrast, elite athletes were reported to be better equipped to handle anxiety, as well as improvement of performance by managing their arousal. ^[17]^ The findings of the current study provides snapshots of how symptoms of anxiety, if not addressed, might result in an increase in the incidence rate of depression and stress symptoms. However, there was a difference when DASS-21 was compared between recreational athletes and elite athletes. ^[17]^ To the best of our knowledge, this is the first study to investigate athlete burnout among semi-professional cricket players in South Africa, as mental health profiling among cricket players is largely unknown. ^[1,3, 4]^

Furthermore, the sub-scales had no correlation with age. Pillay et al. ^[2]^ reported that one in every two athletes was sad, suffering from energy loss and a lack of drive to train. In contrast, this study found 40% of cricket players felt that they do not always reach their maximum athletic potential (i.e. in cricket). In addition, the RSA reported by Hughes attributed to injuries and this might be a rare occurrence in the current study. ^[18]^ Mood illnesses, suicidal thoughts, and substance misuse have all been linked to cricket-specific concerns. ^[1, 4]^ According to the SWLS in this study, cricket players reported a moderate level of life satisfaction. Since there are no clinical cut-off scores for burnout measures, it is also still unknown how many athletes are suffering from this phenomena. ^[19]^

### The impact of COVID-19 on the mental health of cricket players

Training alone and lack of sport-specific training during lockdown led to an increase in mental health symptoms. ^[1]^ Research has identified that COVID-19 increased the phenomenon of mental health disorders, which was evident in an increase in mental fatigue and depression in football. ^[6]^ Ali and Green ^[17]^ found a strong correlation between DASS–21 factors, age and ethnicity; however, the present study found contradictory results.

During lockdown, players were required to be in bio-bubbles and no interaction with people outside of the bio-bubble. As a result, this led to rumination and depressive symptoms. ^[2]^ The current study highlights the effects of COVID-19 on the player’s mental health as some experienced symptoms of depression. These results compliment the findings by Pillay et al. ^[2]^ which depicted symptoms of depression being high during COVID-19. It was further reported by Schuring that there was a link between unhappiness and a significantly elevated risk of distress, anxiety, and depression among cricket players. ^[20]^ It is, therefore, imperative for cricket clubs to be aware of depressive symptoms among cricket players and provide assistance when needed.

### Future research directions for mental health in cricket (and sport in general)

More research is needed to discover the root causes of athletes' discontent with sports so that targeted remedies can be developed. ^[5, 20]^ There are also limited studies investigating SWLS among semi-professional cricket players and the current study provides a snapshot of what transpired during COVID-19. Future research studies should compare the mental health of male and female cricket players respectively to determine whether there are any relationships with their performance. Lastly, research should also be conducted among larger sample groups and players with varied skill levels.

### Limitations

Male cricket players, who are known to be less receptive to mental health questions, were the only participants. Self-reported assessments may provide outcomes that are distorted or subjective. This study could not account for a variety of characteristics, such as the history of mental health difficulties and current treatment, or the direct impact of the COVID-19 outbreak (i.e. bereavement, loss of income, etc.). The generalisability of the conclusions are constrained by the sample of this population, and larger samples are necessary to acquire a deeper understanding of semi-professional cricket players. Due to possible selection bias, the recruited sample may not be typical of semi-professional cricketers in Cape Town, South Africa, and external validity may be limited.

### Recommendations and future studies

A key recommendation from this study is that teams of cricket players should do weekly mental health monitoring to determine whether players require time with a psychologist. The cricket community should consider employing a Mental Health consultant who focuses entirely on the psychological aspects of players, since this would result in specialised sessions for cricketers and enhanced performance. This may also be conducted in collaboration with biokineticists, physiotherapists, and other allied health specialists in order to assist the cricketer holistically. Providing a space where any player may speak openly about their mental health is also paramount.

## Conclusion

The current study provided a snapshot of the prevalence of mental health symptoms experienced by cricket players during COVID-19. The study found that there were moderate levels of anxiety, a reduced sense of achievement and a neutral feeling towards satisfaction with life. Reducing burnout, mental health symptoms and life dissatisfaction would extend the playing careers of cricket players. Destigmatising mental health may result in more robust and accurate self-reports of mental health illnesses among elite athletes, which can enable sustainable change. Existing studies of the psychological aspects among cricket players cannot be compared to post-lockdown situations, as there are currently no comparative or baseline data.

## Figures and Tables

**Table 1:** Combined results from the mental health survey instruments of male semi-professional cricket players (n=90)

**DASS–21 questionnaire responses**				

**Item**	**Score**			
Depression	4.37 ± 5.97			
Anxiety	5.03 ± 6.37			
Stress	6.82 ± 6.48			

**The relationship between different sub-scales of DASS–21 and age**				

	**Age**	**Stress**	**Anxiety**	**Depression**
**Age**	1			
**Stress**	0.058	1		
**Anxiety**	− 0.168	0.79**	1	
**Depression**	− 0.052	0.77**	0.75**	1

**Athlete Burnout Questionnaire (ABQ) responses**				

**Item**	**Score**			
PEE	2.17 ± 1.03			
DSP	1.89 ± 1.06			
RSA	2.99 ± 0.97			

**Satisfaction with Life Scale (SWLS) questionnaire responses**				

**Item**				**Score**
In most ways my life is close to my ideal				4.41 ± 1.39
The conditions of my life are excellent				4.77 ± 1.43
I am satisfied with my life				4.74 ± 1.75
So far, I have gotten the important things I want in life				4.51 ± 1.53
If I could live my life over, I would change almost nothing				3.89 ± 1.94

Data are expressed as mean ± SD where applicable.

** Correlation is significant at the 0.01 level (2-tailed).

PEE, Physical and Emotional exhaustion; DSP, Devaluation of sports practice; RSA, Reduced sense of achievement. Scores: Strongly agree = 7; Agree = 6; Slightly agree = 5; Neither agree nor disagree = 4; Slightly disagree = 3; Disagree = 2; Strongly disagree = 1.
